# Analgosedation During the Use of Non-Invasive Respiratory Supports: A Synthesis of Clinical Evidence and Best Practices

**DOI:** 10.3390/jcm15093418

**Published:** 2026-04-29

**Authors:** Giovanni Misseri, Matteo Piattoli, Alice Mirasola, Cesare Gregoretti

**Affiliations:** 1 Department of Anaesthesia and Intensive Care, Fondazione Istituto “G.Giglio” Cefalù, 90015 Palermo, Italy; c.gregoretti@gmail.com; 2 Department of Anaesthesia and Intensive Care, Umberto I Policlinico di Roma, 00161 Rome, Italy; m.piattoli91@gmail.com; 3 Saint Camillus International University of Health and Medical Sciences “UniCamillus”, 00131 Rome, Italy; alice92m@gmail.com; 4 Department of Anaesthesia, San Giovanni di Dio Hospital, 92100 Agrigento, Italy

**Keywords:** analgesia, intensive care, non-invasive ventilation, sedation

## Abstract

Non-invasive respiratory support (NRS) has become a cornerstone in the management of acute respiratory failure (ARF), offering an alternative or a bridge between conventional oxygen therapy (COT) and invasive mechanical ventilation (iMV). While NRS techniques—including non-invasive ventilation (NIV), continuous positive airway pressure (CPAP), and high-flow nasal oxygen (HFNO)—have demonstrated efficacy in reducing intubation rates and improving outcomes, patient tolerance and synchrony remain critical determinants of success. Analgosedation, the strategic use of analgesics and sedatives, has emerged as an important adjunctive therapy to optimise NRS delivery, reduce patient–ventilator asynchrony, and improve comfort. However, the delicate balance between adequate sedation and the preservation of spontaneous breathing, airway protection, and hemodynamic stability presents unique challenges. This comprehensive narrative review synthesises current evidence on analgosedation strategies during NRS use, examining pharmacological agents, their pharmacokinetic and pharmacodynamic properties, comparative studies, indications, monitoring parameters, clinical settings, and safety considerations. We also review existing guidelines, discuss special considerations in paediatric populations, and propose practical clinical approaches. Understanding the nuanced application of analgosedation is essential for clinicians to maximise therapeutic benefit while minimising risks of NRS treatment failure and adverse outcomes.

## 1. Introduction

Non-invasive respiratory support (NRS) encompasses a spectrum of ventilatory strategies that provide respiratory assistance while avoiding endotracheal intubation and its potentially deleterious consequences. These modalities include non-invasive ventilation (NIV), which delivers positive pressure ventilation through interfaces such as facial or nasal masks; continuous positive airway pressure (CPAP), which maintains a constant positive airway pressure throughout the respiratory cycle; and high-flow nasal oxygen (HFNO), which delivers heated and humidified oxygen at high flow rates [1,2,3]. Over the past three decades, NRS has revolutionised the management of acute respiratory failure (ARF) across diverse aetiologies, including acute hypercapnic respiratory failure in chronic obstructive pulmonary disease (COPD), cardiogenic pulmonary oedema, immunocompromised states, and de novo acute hypoxemic respiratory failure (de novo AHRF) [4,5]. The success of NRS hinges upon multiple factors, with patient tolerance and adherence being paramount [6,7]. Discomfort related to the applied interface, anxiety, dyspnoea, and patient–ventilator asynchrony are common barriers that can lead to NRS failure and subsequent need for invasive mechanical ventilation (iMV) [8]. In this context, analgosedation—the judicious application of analgesic and sedative medications—has been proposed as a strategy to enhance NRS tolerance and efficacy. However, the use of sedation in spontaneously breathing patients receiving NRS presents a clinical paradox: while sedation may improve comfort and synchrony, excessive sedation can suppress respiratory drive, compromise airway protection, and precipitate respiratory failure [9]. When considering the goals of sedation for intensive care unit (ICU) patients, these are to provide analgesia and comfort, preserving day/night cycles and avoiding nuisances such as ambient light and noise. Appropriate analgosedation protocols should also include additional goals, such as haemodynamic stability, preservation of metabolic homeostasis, muscular relaxation, preservation of diaphragmatic function and attenuation of the stress/immune response, and considerations such as programmed withdrawal from sedation. These goals should also be achieved in patients while on NRS treatment [10].

Despite the growing interest in analgosedation for NRS, clinical practice remains heterogeneous, and evidence-based guidance is limited [3]. According to recent estimates, less than 20% of patients on NRS receive analgosedation: when used alone, analgesics and/or sedatives show no apparent effects on clinical outcomes (e.g., the failure of NRS and need for iMV); however, the simultaneous use of different drugs has been associated with higher risks of NRS failure [11]. The 2015 Cochrane review on this topic identified only six randomised controlled trials with significant methodological limitations, highlighting the paucity of high-quality evidence [2]. Nonetheless, increasing observational data and recent trials have begun to evaluate the potential risks and benefits associated with various analgosedative agents in this setting. This comprehensive narrative review aims to synthesise the available evidence, provide practical guidance, and identify knowledge gaps to inform future research and clinical practice.

## 2. Materials and Methods

This narrative review was conducted to provide a comprehensive synthesis of the current literature on analgosedation during NRS. The review aimed to provide a clinically oriented overview of indications for analgosedation during NRS, pharmacological options, monitoring strategies and safety considerations, and suggested clinical approaches. For these purposes, special attention was given to physiological effects of sedatives, their impact on NRS tolerance and success, need for endotracheal intubation, safety profile and adverse events, and practical aspects of analgosedation strategies. A systematic search strategy was employed to identify relevant studies published in peer-reviewed journals. We searched PubMed/MEDLINE, Embase, Cochrane Central Register of Controlled Trials (CENTRAL), and Web of Science from inception through March 2026. Search terms included combinations of the following keywords and Medical Subject Headings (MeSH) terms: “non-invasive ventilation”, “NIV”, “non-invasive respiratory support”, “NRS”, “CPAP”, “high-flow nasal oxygen”, “HFNO”, “sedation”, “analgosedation”, “analgesia”, “dexmedetomidine”, “remifentanil”, “propofol”, “midazolam”, “morphine”, and “benzodiazepines”. Boolean operators (AND, OR) were used to combine search terms appropriately. Inclusion criteria comprised (1) studies involving adult and paediatric patients receiving NRS (NIV, CPAP, or HFNO); (2) studies evaluating analgosedative interventions; (3) randomised controlled trials, observational studies, systematic reviews, and meta-analyses; and (4) studies published in English. Exclusion criteria included (1) studies focusing exclusively on invasive mechanical ventilation, (2) case reports with fewer than five patients, and (3) studies not directly addressing analgosedation strategies. Two independent reviewers screened titles and abstracts for eligibility, followed by full-text review of potentially relevant articles. Studies were selected based on their relevance to the topic. Reference lists of included studies and relevant review articles were manually searched to identify additional studies. When multiple studies addressed similar interventions, their findings were compared qualitatively to identify consistent patterns, controversies, and gaps in the literature. Both adult and paediatric data were considered where relevant, with emphasis on studies conducted in critical care settings. Given the heterogeneity of study designs, interventions, and outcome measures, a narrative synthesis approach was adopted rather than quantitative meta-analysis.

## 3. Risk Factors and Predictors of NRS Failure

Understanding the risk factors and predictors of NRS failure is crucial for identifying patients who may benefit from analgosedation. NRS failure, typically defined as the need for endotracheal intubation and escalation to iMV, occurs in up to 40% of patients depending on the underlying aetiology, disease severity, and clinical setting [12,13,14]. Patient-related factors associated with increased risk of NRS failure include advanced age, higher illness severity scores (APACHE II > 29, SAPS II > 35), altered mental status at initiation, severe acidosis (pH < 7.25), profound hypoxemia (PaO_2_/FiO_2_ ratio < 150), and excessive secretions [15,16]. The presence of pneumonia, particularly hospital-acquired or ventilator-associated pneumonia, carries a higher failure rate compared to COPD exacerbations or cardiogenic pulmonary oedema [17]. Obesity with body mass index > 35 kg/m^2^ has been identified as both a risk factor for failure and a predictor of difficult mask fitting and air leakage. Physiological parameters measured during the first hours of NRS application have strong predictive value. Persistent tachypnoea (respiratory rate > 35 breaths/minute) after one to two hours of NRS, inability to reduce PaCO_2_ levels by at least 20% in COPD patients, and worsening or lack of improvement in pH are associated with subsequent failure [12,15]. Patient–ventilator asynchrony, manifested as ineffective triggering, double triggering, or autotriggering, predicts poor outcomes and may be amenable to intervention through improved settings or judicious sedation [18]. Interface-related issues constitute a major category of NRS failure. Intolerance due to claustrophobia, discomfort, skin breakdown, or air leakage accounts for 10% to 30% of treatment discontinuations [18,19]. Excessive agitation or anxiety, whether related to dyspnoea, the underlying disease process, or the interface itself, significantly compromises the effectiveness of NRS. A 2025 systematic review found dyspnoea present in 40.6% (95% CI, 37.8–43.5) of communicative mechanically ventilated patients, with moderate to severe intensity [20]. In the case of NRS use, dyspnoea affects similar proportions of patients. A 2018 study of 426 patients receiving NIV for acute respiratory failure found median dyspnoea scores of 4/10 on admission, decreasing to 3/10 after the first NIV session [21]. Importantly, dyspnoea intensity ≥ 4 after the first NIV session was independently associated with NIV failure (OR 2.41) and mortality (OR 2.11). Sleep disruption was identified as a concomitant factor in late NIV failure [22]. In this case, an important role is played by reversible environmental causes (i.e, noise and disturbance), which should be addressed before proceeding with the use of sedatives. It has been demonstrated that ventilator asynchrony may disrupt physiologic sleep patterns, and evidence suggests that sleep is associated with higher asynchrony-index values and blood-oxygen desaturations when compared to awake patients [23]. All the above-mentioned factors represent potentially modifiable targets for analgosedative intervention. Several prediction models have been developed to stratify risk. The HACOR score (Heart rate, Acidosis, Consciousness, Oxygenation, Respiratory rate), when measured one hour after NIV initiation, demonstrates good discriminative ability with an area under the ROC curve of 0.89 for predicting NIV failure [24]. Scores greater than 5 points indicate high risk. Similarly, the ROX index (ratio of SpO_2_/FiO_2_ to respiratory rate) has emerged as a useful predictor in patients receiving HFNO, with values below 3.85 at 2–6 h associated with increased likelihood of HFNO failure and need for intubation [25]. Serial measurements of the ROX index are recommended, as trends over time provide additional prognostic information. These scoring systems can help identify patients who might benefit from closer monitoring, optimisation of NRS parameters, or consideration of analgosedation to improve tolerance and outcomes.

## 4. Pharmacological Agents for Analgosedation During NRS Use

### 4.1. Pharmacokinetics and Pharmacodynamic Properties of Analgosedatives Agents

The ideal analgosedative agent for use during NRS would provide anxiolysis and comfort without suppressing respiratory drive, maintain hemodynamic stability, allow rapid titration to desired effect, and enable quick recovery upon discontinuation. Unfortunately, no single agent perfectly fulfils all these criteria. Therefore, careful selection based on individual patient characteristics and clinical circumstances is needed.

Dexmedetomidine is a highly selective alpha-2 adrenergic agonist that has garnered significant attention for use during NRS. Its unique pharmacological profile includes sedation without significant respiratory depression, preservation of arousability, and moderate analgesic properties. Dexmedetomidine has a distribution half-life of approximately 6 min and an elimination half-life of 2 to 3 h, allowing for relatively predictable offset of effects. The drug is metabolised hepatically via glucuronidation and cytochrome P450 pathways, requiring dose adjustment in severe hepatic impairment. The onset of sedation occurs within 15 min of loading dose administration, with peak effects at 15–30 min [26]. Notably, dexmedetomidine-induced sedation occurs through activation of alpha-2 receptors in the locus coeruleus, producing a sedative state that resembles natural sleep and allows patients to be easily aroused, which is particularly advantageous when cooperation is required during NRS. The primary adverse effects of dexmedetomidine are hemodynamic: bradycardia and hypotension due to reduced sympathetic outflow and increased vagal activity. A biphasic blood pressure response may occur with initial transient hypertension from peripheral alpha-2B receptor activation, followed by hypotension from central alpha-2A effects. These cardiovascular effects can be minimised by avoiding rapid loading doses and maintaining infusion rates below 0.7 mcg/kg/hour [27]. Dexmedetomidine does not cause significant respiratory depression at clinical doses, making it theoretically well-suited for NRS application where preservation of spontaneous ventilation is critical. Dexmedetomidine also exerts a lung-protective effect by acting on pulmonary vascular resistances (via a dose-dependent change in blood catecholamine levels), pulmonary ischemia-reperfusion injury, and inflammatory factor release [28].

Opioids are universally used in mechanically ventilated critically ill patients for analgosedation. Beyond their analgesic and sedative properties, opioids exert significant effects on respiratory muscle tone and breathing pattern. Remifentanil is an ultra-short-acting opioid analgesic with unique pharmacokinetic properties. Unlike other opioids that undergo hepatic metabolism, remifentanil is rapidly metabolised by nonspecific plasma and tissue esterases, resulting in a context-sensitive half-time of approximately 3–4 min regardless of infusion duration. This predictable offset makes remifentanil one of the most promising drugs for analgosedation in critically ill patients. It provides potent analgesia and some degree of anxiolysis, with onset of action within 1–2 min and peak effect at 2–3 min. However, remifentanil shares the respiratory depressant effects common to all mu-opioid receptor agonists, including dose-dependent reduction in respiratory rate and minute ventilation, decreased responsiveness to carbon dioxide, and the potential for apnoea at higher doses. This respiratory depression can paradoxically compromise the very indication for NRS. The analgosedative effects of remifentanil on respiratory drive and timing varies depending on doses applied: it has been demonstrated that opposed to propofol, remifentanil prolongs patient’s expiratory time at doses exceeding 0.05 mcg/kg/min while not affecting respiratory drive [29]. Additionally, opioids can cause chest wall rigidity at high doses, nausea, pruritus, and the development of acute tolerance with prolonged infusions. Despite these concerns, low-dose remifentanil infusions (0.025–0.1 mcg/kg/min) have been studied in NRS settings with careful monitoring [30]. These showed an improvement in gas exchanges (decrease in PaCO2 and increase in PaO2/FiO2 ratio) in agitated patients with AHRF under NIV [31]; this is probably due to greater patient comfort with less patient–ventilator asynchrony. An experimental study also showed that remifentanil could improve respiratory pattern and decrease inspiratory muscle effort, while not affecting oxygenation or sedation targets [31]. In addition, opioids are the most evidence-based agents for dyspnoea relief. Morphine and remifentanil are effective in reducing breathlessness without causing clinically significant respiratory depression in patients receiving NIV. According to the European Respiratory Society/European Society of Intensive Care Medicine, opioids are the mainstay for dyspnoea that persists despite optimal treatment of the underlying cause: an initial intravenous titration with immediate-release opioids until dyspnoea relief is achieved, followed by continuous low-dose administration, is a commonly employed strategy [32].

Propofol is a GABA-A receptor agonist that produces rapid-onset sedation and amnesia. With an onset of action within 30–45 s and distribution half-life of 2–4 min, propofol allows for precise control of sedation depth. However, its context-sensitive half-time increases with prolonged infusion due to redistribution from peripheral compartments. Propofol undergoes hepatic metabolism and requires dose reduction in the presence of hepatic dysfunction [33]. The major limitations of propofol when applied to NRS relate to its profound respiratory and hemodynamic depressant effects: propofol causes dose-dependent respiratory depression; therefore, special care must be taken for its potential adverse effects on respiratory drive and upper airway patency [34], making it unsuitable for most NRS applications where preservation of spontaneous breathing is essential. Hypotension results from decreased systemic vascular resistance, myocardial depression, and reduced sympathetic tone. For these reasons, propofol is generally reserved for short procedural sedation during NRS or for facilitating transition to iMV when failure of NRS appears imminent. Propofol target-controlled infusion (TCI) should be preferred to reduce its adverse effects often associated with drug accumulation. This is a method of administering anaesthetic intravenous agents based on their pharmacokinetic profile, using a computerised algorithm protocol, which allows rapid and accurate drug plasmatic concentration [35]. Unfortunately, TCI cannot be used for prolonged periods due to a loss of accuracy.

The analgosedative effects of ketamine might be useful in patients during NRS treatment. However, this drug is difficult to titrate effectively to prevent its common adverse reactions, including excessive salivation, delirium, and increased sympathetic nervous system stimulation [36].

Benzodiazepines (primarily midazolam and lorazepam) act on GABA-A receptors to produce anxiolysis, sedation, and amnesia. However, accumulation of active metabolites, particularly in cases of renal insufficiency, can prolong their effects in an unpredictable fashion. Additional concerns include dose-dependent respiratory depression and increased risk of delirium [37]. The 2013 Pain, Agitation, and Delirium (PAD) guidelines recommend against routine benzodiazepine use for sedation in mechanically ventilated patients, favouring alternatives [38]. This guidance likely extends to NRS settings, where benzodiazepines should be used sparingly and only when specific indications exist.

### 4.2. Comparative Studies, Meta-Analyses, and Randomised Controlled Trials

Although steadily increasing, research supporting analgosedation in NRS remains limited. The 2015 Cochrane systematic review by Clouzeau et al. identified only six randomised controlled trials (RCTs) involving 235 participants, highlighting significant knowledge gaps [2]. This review found insufficient evidence to determine whether sedation during NIV improves comfort or success rates while noting concerns about potential adverse effects, including respiratory depression. Several subsequent RCTs have expanded the evidence base. A landmark study by Huang et al. randomised 90 patients with ARF on NIV to receive either dexmedetomidine infusion or placebo [6]. The dexmedetomidine group demonstrated significantly lower intubation rates (13.3% vs 33.3%, *p* = 0.029), improved patient–ventilator synchrony, and better NIV interface tolerance without increased adverse events. Mean sedation scores remained in the light sedation range, and respiratory depression was not observed. This study provided compelling evidence that carefully titrated dexmedetomidine could improve NIV outcomes in selected patients.

Liu et al. conducted an RCT comparing low-dose remifentanil to a placebo in 52 patients receiving NIV for acute respiratory failure [7,39]. The remifentanil group (0.03–0.05 mcg/kg/min) showed improved NIV tolerance scores and reduced patient–ventilator asynchrony without significant differences in respiratory rate, PaCO_2_, or intubation rates compared to controls. However, the study was underpowered to detect differences in clinical outcomes, and close monitoring was required to avoid respiratory depression.

A randomised trial by Senoglu et al. compared dexmedetomidine to midazolam in 40 patients receiving NIV post-extubation [40]. Dexmedetomidine-treated patients had lower re-intubation rates, better arterial blood gas parameters, and fewer episodes of NIV intolerance compared to midazolam-treated patients. The midazolam group experienced more respiratory depression events and higher rates of NIV discontinuation due to excessive sedation, supporting the preferential use of dexmedetomidine when sedation is indicated.

Devlin et al. performed a pilot RCT comparing dexmedetomidine to a placebo in 20 patients receiving NIV [3]. While the study was limited by a small sample size, it demonstrated the feasibility and safety of dexmedetomidine use, with trends toward improved NIV tolerance and reduced need for rescue sedation. Importantly, no episodes of severe respiratory depression or hemodynamic instability occurred in the dexmedetomidine group.

When three sedative drugs (dexmedetomidine, propofol, and remifentanil) were compared in a prospective, randomised cohort study [41], the use of sedation increased NIV success in patients nontolerant to NIV treatment. Patients treated with dexmedetomidine infusion were found to be more compliant to NIV interfaces and showed lower mortality rates and ICU length of stay.

A meta-analysis by Xu et al. pooled data from eight RCTs (n = 478) comparing dexmedetomidine to control or alternative sedatives during NIV [42]. The analysis found that dexmedetomidine significantly reduced intubation rates (RR 0.59, 95% CI 0.39–0.88, *p* = 0.01), improved NIV tolerance, and decreased patient–ventilator asynchrony. Subgroup analysis suggested greater benefit in patients with moderate-to-severe agitation and in those with COPD exacerbations. The incidence of bradycardia was higher with dexmedetomidine (RR 2.14, 95% CI 1.12–4.10), but this rarely required intervention. No significant differences were found in hypotension, respiratory depression, or mortality. These findings were furtherly confirmed in more recent metanalysis [43]. In addition, the use of analgosedation during NRS application showed to reduce the rate of delirium, endotracheal intubation, and hospital length of stay in different categories of patients with ARF [44].

More recent observational studies have explored analgosedation in specific populations and with novel NRS modalities. A recent retrospective multicenter cohort study on patients with AHRF treated with NIV [45] identified 433,357 patients of whom 26.7% received sedation or analgesia, of which 11.7% received opioids only, 9.4% received benzodiazepines only, 4.6% received opioids and benzodiazepines, 0.4% received dexmedetomidine only, and 0.6% received dexmedetomidine in addition to opioid and/or benzodiazepine. Of 433,357 patients receiving NIV, 50,413 (11.6%) patients underwent iMV after 2 days of hospital admission.

Dexmedetomidine showed promising results in blunt-chest trauma patients treated with NIV [46], as it prolonged the duration of NIV treatment, reducing NIV intolerance and achieving better sedation targets when compared to placebo administration.

A multicenter prospective RCT [47] enrolling 179 adult cardiac surgery patients with moderate-to-severe intolerance to NIV demonstrated that the mitigation rate, defined as the proportion of patients who were relieved from their initial NIV intolerance status, was not significant at most timepoints except for the 15 min timepoint (42% vs. 20%, *p* = 0.002) when comparing remifentanil and dexmedetomidine infusions. This was the first study showing that remifentanil was as effective as dexmedetomidine in analgosedation practices.

Studies examining analgosedation during HFNO are more limited but emerging. Most of these assess procedural sedation protocols, where the application of HFNO is used to guarantee adequate oxygenation. Preliminary data suggest that low-dose dexmedetomidine in combination with opioids may improve HFNO tolerance in selected patients and especially in procedures like ERCP [9]. However, further research is needed to establish optimal sedation protocols.

### 4.3. Clinical Indications for Analgosedation During NRS Treatment

**Figure 1:**
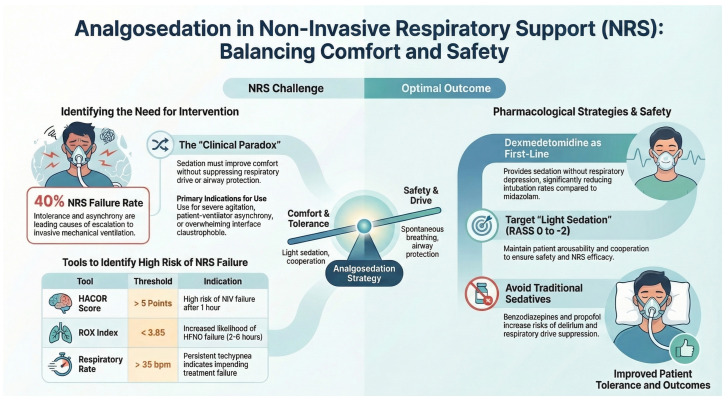
Clinical indications, drugs, and monitoring strategies for analgosedation during non-invasive respiratory support (NRS).

The decision to use analgosedation during NRS must be individualised, weighing potential benefits against risks. Not all patients receiving NRS require or benefit from sedative medications, and inappropriate use can precipitate respiratory failure. Therefore, clear indications help guide appropriate patient selection (Figure 1) [48].

Severe agitation or anxiety that compromises NRS effectiveness despite optimised interface fit, ventilator settings, and non-pharmacological comfort measures are the main indications for analgosedation [8,10,49]. Patients exhibiting restlessness, pulling at the interface, an inability to maintain the mask in position, or fighting the ventilator despite adequate oxygenation and ventilation may benefit from judicious sedation. A lack of compliance to the interface or ventilator settings may stem from dyspnoea, interface-related discomfort, delirium, or the underlying critical illness. Hence, the correct evaluation of reversible causes of discomfort should always be assessed before initiation of analgosedation protocols. Patient–ventilator asynchrony [50] manifests as ineffective triggering, double triggering, premature cycling, or delayed cycling. In order to resolve such issues, the first-line approach should be optimising ventilator parameters (i.e., adjusting trigger sensitivity, inspiratory time, pressure support level, or PEEP). However, persistence of asynchronies despite ventilator setting adjustments may warrant sedation to improve patient–ventilator interaction [8]. Interface intolerance due to claustrophobia or discomfort constitutes a common indication to analgosedation. Some patients, particularly those with no prior NRS experience or those with anxiety disorders, might present overwhelming claustrophobia with mask interfaces. After attempting alternative interface types (nasal masks, total face masks, or helmet interfaces) and apply rotation of the interfaces, mild sedation may facilitate adaptation to NRS [10]. Pain that interferes with NRS tolerance, particularly in patients with thoracic trauma, post-operative states, or uncomfortable positioning requirements (e.g., prone position) [51,52], represents an indication for analgesia. Inadequately treated pain increases sympathetic drive, oxygen consumption, and the work of breathing while reducing compliance to NRS. Opioids can improve comfort and reduce dyspnoea perception, though careful titration is essential to avoid their adverse effects.

Prophylactic sedation to facilitate transition to NIV following planned extubation in high-risk patients (such as those with difficult airways, obesity, or previous extubation failure) has also been explored [53]. The rationale is that maintaining light sedation during the critical post-extubation period may improve NIV tolerance and prevent re-intubation. However, this approach requires careful patient selection and monitoring, as over-sedation can negatively affect the benefits of early extubation.

Lastly, clinicians should also consider several situations representing contraindications to analgosedation during NRS [9,10,54]. These include absent or minimal respiratory drive, a severely depressed level of consciousness (Glasgow Coma Scale < 8–10), hemodynamic instability requiring vasopressor support, an inability to protect the airway with high aspiration risk, and copious secretions requiring frequent suctioning. In these cases, the risks of analgosedation outweigh potential benefits, and consideration should be given to iMV if application of NRS is not feasible and respiratory support is needed.

## 5. Monitoring and Safety Considerations

Monitoring of vital parameters, sedation, and pain intensity is imperative when administering analgosedation during NRS to balance therapeutic benefits with the inherent risks of respiratory depression, hemodynamic instability, and over-sedation. The monitoring strategy must be multimodal, continuous, and interpreted by experienced clinicians able of rapidly responding to deterioration [24,55]. The level of consciousness and sedation depth require systematic assessment using validated scales. The Richmond Agitation–Sedation Scale (RASS) [56] is widely used and allows for standardised communication among providers. For patients receiving analgosedation during NRS, target sedation levels typically range from 0 (alert and calm) to −2 (light sedation), avoiding deeper sedation that compromises arousability. The Riker Sedation–Agitation Scale (SAS) [57] represents an alternative. Regular RASS or SAS scoring (every 1–2 h at minimum, more frequently during titration) helps prevent over-sedation and guides dose adjustments. Hemodynamic monitoring includes continuous electrocardiography and frequent (at least every 15 min) blood pressure (noninvasive or invasive) measurements. When using dexmedetomidine, vigilance for bradycardia and hypotension is required. Healthcare providers must pay careful attention to respiratory mechanics, respiratory rate evaluation, and the possible recruitment of accessory respiratory muscle. Arterial blood gas analyses should be performed at baseline, within 1–2 h of initiating analgosedation, and subsequently based on clinical status. Serial blood gases allow for the assessment of gas exchange trends. In patients with baseline hypercapnia (COPD, obesity hypoventilation), particular attention must be paid to PaCO_2_ trends. Ventilator parameters, flow and pressure curves, and expiratory tidal volume (when possible) must be monitored. Patient comfort and NIV tolerance should be assessed using visual analogue scales or numeric rating scales. Several NIV-specific comfort scales have been validated, such as the NIV-SAT (NIV-Specific Comfort Scale) [8]. Regular assessment of patient-reported outcomes helps guide dose titration and identifies situations where technical adjustments might be more appropriate than increased sedation. Monitoring for interface-related complications remains important even when sedation is used. Skin breakdown, particularly over the nasal bridge, can develop rapidly. Regular interface repositioning, rotation of interfaces, and use of protective dressings can mitigate this risk [49]. Eye irritation from air leaks should be assessed, as corneal abrasions can occur with excessive leakage toward the eyes. Gastric distension from air swallowing may cause discomfort and increase aspiration risk, warranting nasogastric tube placement in some cases. The nursing ratio must be appropriate for the level of monitoring required: patients receiving continuous infusions of analgosedatives during NRS typically require 1:1 or 1:2 nurse-to-patient ratios in intensive care or high-dependency settings. Staff should be trained in NRS management, sedation assessment, and emergency airway management [58]. Outside of these settings, experiences of analgosedation during NRS are reported, especially in sub-intensive care units [17], in emergency departments [59], in post-anaesthesia recovery rooms [60], and in hospital wards. However, it is recommended that analgosedation protocols be applied when adequate monitoring and trained healthcare providers are present [9,54].

## 6. Current Guidelines and Recommendations on Analgosedation Strategies During NRS Treatment

Clinical practice guidelines specifically addressing analgosedation during NRS remain sparse. However, several relevant guidelines and expert consensus documents provide context and recommendations. Recent expert opinion papers have advocated for a more nuanced approach to analgosedation during NRS. These suggest that rather than categorically avoiding sedation, clinicians should employ individualised risk–benefit assessment, recognise clinical scenarios where analgosedation may be beneficial, select appropriate agents and doses, and implement robust monitoring protocols. This evolution in thinking reflects growing clinical experience and emerging evidence supporting carefully applied analgosedation protocols in selected patients.

The 2017 Clinical Practice Guidelines for the Prevention and Management of Pain, Agitation/Sedation, Delirium, Immobility, and Sleep Disruption in Adult Patients in the ICU (PADIS guidelines) from the Society of Critical Care Medicine do not specifically address NRS but provide general principles applicable to critically ill patients [38]. The guidelines recommend systematic assessment of pain and sedation using validated tools, preferential use of non-benzodiazepine sedatives (specifically dexmedetomidine or propofol over benzodiazepines) in mechanically ventilated adults, and multimodal analgesia strategies. These recommendations might be extended to NRS settings.

The European Respiratory Society/American Thoracic Society clinical practice guideline on NIV for acute respiratory failure (2017) acknowledges that patient comfort and synchrony are critical for NIV success but does not make specific recommendations about analgosedation [61]. The guideline notes that “sedation should be used with extreme caution during NIV, as it may precipitate respiratory failure,” and it emphasises that non-pharmacological interventions should be prioritised first. When sedation is deemed necessary, the guideline suggests selecting agents with minimal respiratory depressant effects and implementing intensive monitoring.

The British Thoracic Society guideline on NIV in acute respiratory failure (2016) similarly takes a cautious approach, stating that sedation during NIV is generally not recommended but may be considered in exceptional circumstances for agitated patients when NIV is failing due to patient–ventilator asynchrony [62]. The guideline recommends that if sedation is required, it should be administered only by experienced practitioners in closely monitored environments, preferably in ICUs.

The Canadian Clinical Practice Guidelines for NIV in acute respiratory failure (2011) provide more specific guidance, recommending consideration of sedation for select patients with severe agitation or intolerance despite optimisation of NIV settings and interfaces [7]. The guidelines suggest short-acting agents, and dexmedetomidine may be preferable to benzodiazepines or propofol in this setting, though evidence was limited at the time of guideline development.

Recent published expert recommendations on NRS for the treatment of ARF in the ICU include the following: (1) sedation during NIV should be avoided when possible; (2) non-pharmacological measures (coaching, interface optimisation, environmental modifications) should be exhausted before pharmacological sedation; (3) when sedation is required, target light sedation (RASS 0 to −2); (4) dexmedetomidine appears to be the safest sedative option based on available evidence; (5) benzodiazepines and propofol should generally be avoided; and (6) opioids may be appropriate for patients with pain or severe dyspnoea but require careful respiratory monitoring [9,63]. In 2024, the Italian Society of Anaesthesia, Analgesia, and Intensive Care (SIAARTI) [9,54] developed eight best clinical practice statements intended to provide the basis for building a decision-making process determining an individualised approach for the application of analgosedation protocols during NRS use. According to the panel’s opinion, it is essential to understand the underlying clinical condition in order to determine patients who could benefit from analgosedation during NRS, the characteristics of the analgesic and/or sedative drugs available, the type of monitoring and the clinical setting where to start such protocols. The panel concludes that application of analgosedation during NRS treatment should be considered when indicated and applied by well-trained healthcare staff and in appropriate clinical settings. Table 1 presents a practical clinical framework of analgosedation application during NRS treatment.

## 7. Analgosedation During NRS Use in Paediatric Patients

Age-specific considerations profoundly influence the approach to analgosedation in the paediatric population. Infants and young children have greater susceptibility to respiratory depression from sedatives due to immature respiratory control mechanisms, smaller functional residual capacity, and higher metabolic demands. Conversely, older children and adolescents may experience significant anxiety and claustrophobia similar to adults. The ability to cooperate and communicate varies widely across paediatric age groups, necessitating individualised strategies. Evidence for analgosedation during paediatric NRS is even more limited than in adults, consisting primarily of small observational studies and case series. A systematic review by Piastra et al. identified only three small studies examining sedation during paediatric NIV, with highly variable practices and insufficient data to draw firm conclusions [64]. The review highlighted that while sedation may improve NIV tolerance in some children, this must be carefully weighed against the risks of respiratory depression and NIV failure. Non-pharmacological strategies assume even greater importance in paediatric NRS. These include gradual interface acclimation during a calm period, allowing the child to hold the mask at first, the use of age-appropriate distraction techniques (videos, games, music), parental presence and coaching, and the involvement of child-life specialists. Proper interface selection and sizing are critical, as interfaces specifically designed for paediatric use with minimal dead space and good seal are essential. Many paediatric programs have developed desensitisation protocols where children practice with the NRS interface during wake periods to reduce fear and improve cooperation. When pharmacological interventions are necessary, dexmedetomidine has emerged as the preferred agent [65]. Multiple case series have reported successful use of dexmedetomidine to facilitate NIV tolerance in children. Typical dosing ranges from 0.2 to 0.7 mcg/kg/hour without loading dose or with small loading doses (0.5 mcg/kg over 10 min) in older children. The preservation of respiratory drive, maintenance of arousability, and relatively favourable safety profile make dexmedetomidine attractive for paediatric applications [66,67,68]. A retrospective review of 40 children receiving dexmedetomidine during NIV found improved patient comfort scores and NIV synchrony with no episodes of severe respiratory depression [69]. A more recent study by Eidman et al. examined 60 children receiving NIV with dexmedetomidine sedation and reported increased tolerance to NIV and no difference in intubation rate at 6 h between subjects receiving dexmedetomidine versus no sedation (13.1 vs. 12.4%) [65]. Monitoring requirements in paediatric patients mirror those in adults but may require age-appropriate modifications. Continuous pulse oximetry, frequent respiratory rate assessment, and regular sedation scoring using paediatric-specific scales (such as the COMFORT scale or State Behavioural Scale) are standard [70,71]. The threshold for escalation to invasive ventilation may be lower in young children given their limited respiratory reserve. Family involvement in monitoring for changes in child’s responsiveness or breathing pattern can provide an additional layer of safety.

## 8. Knowledge Gaps and Future Research Directions

The application of NRS has transformed the management of acute respiratory failure, and its success depends critically on patient tolerance and adherence. While avoiding the complications associated with iMV, it introduces unique challenges related to interface comfort, patient–ventilator synchrony, and anxiety management. Analgosedation represents a double-edged intervention in this context—capable of enhancing NRS effectiveness in appropriately selected patients while possibly increasing treatment failure when misapplied [72]. Evidence supporting analgosedation during NRS, though expanding, remains limited: Current data suggest that carefully selected and dosed sedative agents, particularly dexmedetomidine, can improve NRS tolerance and reduce intubation rates. Dexmedetomidine’s unique pharmacological profile—providing sedation without significant respiratory depression while preserving arousability—makes it the most promising agent for this application. Conversely, traditional sedatives like benzodiazepines and propofol carry substantial risks and should be avoided in most cases. The decision to use analgosedation during NRS must be personalised and preceded by optimisation of non-pharmacological interventions. Technical factors, including interface selection and fit, ventilator parameters optimisation, environmental modifications, and behavioural strategies, should be addressed first. When pharmacological interventions become necessary, clear indications should be present, and appropriate agents should be selected focusing on light sedation. Special consideration must be given to vulnerable populations, particularly paediatric patients, where evidence is even more limited and developmental factors profoundly influence both the need for and risks of sedation. Current guidelines appropriately emphasise caution, recommending that sedation during NRS be used selectively rather than routinely. However, the evolution of clinical practice and emerging evidence suggest that categorical avoidance of sedation may deny potential benefits to certain patients. High-quality randomised controlled trials are needed to define optimal patient selection criteria, compare sedative agents head-to-head, establish dose–response relationships, identify biomarkers or clinical parameters that predict benefit, and determine long-term outcomes. The role of analgosedation in specific NRS modalities—particularly HFNO—requires further investigation. Studies examining sedation strategies in specific populations (immunocompromised patients, patients affected from obesity hypoventilation syndrome—OHS, the use of NRS in post-extubation respiratory failure prophylaxis) would inform practice in these areas.

## 9. Conclusions

Analgosedation during NRS represents a valuable but complex intervention requiring careful clinical judgment. When applied appropriately in selected patients with clear indications, using agents with favourable safety profiles, targeting light sedation, and implementing comprehensive monitoring, analgosedation can enhance NRS tolerance and success. Future research must address persistent knowledge gaps to enable more evidence-based and personalised approaches.

## Figures and Tables

**Table 1:** Practical clinical framework for analgosedation during non-invasive respiratory support (NRS).

Section	Domain	Key Actions/Criteria
**Patient selection**	Indications	Severe agitation despite non-pharmacological measures Significant patient-ventilator asynchrony after technical optimization Interface intolerance limiting NRS effectiveness Pain interfering with respiratory therapy
	Baseline assessment	Consciousness, RR and pattern, SpO_2_, BP/HR, ABG; calculate HACOR score and ROX index
	Documentation	Record specific indication and anticipated therapeutic goals
**Non-pharmacological optimisation**	Interface	Correct sizing; switch mask type (oronasal → nasal → helmet); protective cushions
	Ventilator settings	Optimise trigger sensitivity, PS/PEEP, rise time, cycling criteria, ventilator mode
	Environment and behavior	Reassurance, scheduled interface breaks, positioning, noise/light reduction, day-night cycles, family involvement; age-specific distraction in paediatrics
**Agent selection & initial dosing**	First line – sedation Dexmedetomidine	Start 0.2–0.4 mcg/kg/h (no loading dose); optional slow load 0.5–1.0 mcg/kg over 10–20 min in stable patients, followed by maintenance
	Pain/dyspnoea Opioids	Low-dose remifentanil 0.025–0.05 mcg/kg/min or morphine 1–3 mg IV bolus q15–30 min, only with adequate respiratory drive and close monitoring
	Avoid/last resort Benzodiazepine/propofol	Avoid routinely; midazolam 0.5–1 mg IV bolus only if alternatives have failed, with intensive monitoring
**Titration and targets**	Sedation target	RASS 0 to −2 (light sedation, patient arousable and cooperative)
	Titration protocol	Dexmedetomidine: ±0.1–0.2 mcg/kg/h every 20–30 min; reassess every 15–30 min during titration, then hourly; reduce dose for excessive sedation, respiratory depression, HR < 50, SBP < 90 or MAP < 65 mmHg
**Monitoring**	Continuous	Pulse oximetry, ECG/HR display, capnography (if feasible)
	Intermittent	BP q15–30 min (then hourly when stable); RR and pattern; RASS/SAS; comfort/tolerance scales; ABG at baseline, 1–2 h after initiation, then q4–6 h or with any clinical change
	Alarm thresholds and staffing	SpO_2_ < 88–90%; HR < 50 or >130 bpm; apnoea alert if available; nurse ratio 1:1 or 1:2; staff trained in NRS, sedation assessment, emergency airway, reversal agents and code cart immediately available
**Troubleshooting and de-escalation**	Excessive sedation/respiratory depression	Reduce or hold infusion; increase monitoring; ensure oxygenation via NRS or bag-mask; consider reversal (naloxone, flumazenil); prepare for intubation if deterioration continues
	Haemodynamic complications (dexmedetomidine)	Bradycardia: reduce/hold if HR < 40–50 or symptomatic; anticholinergics for persistent cases; HR 50–60 usually tolerable if asymptomatic Hypotension: fluid bolus if appropriate, reduce/cease infusion, vasopressor support if needed
	De-escalation	Daily sedation interruption/weaning trial once indication resolves; taper gradually (avoid abrupt dexmedetomidine withdrawal); reassess need daily
**Recognising failure and transition**	Intubation indications	Worsening gas exchange (progressive hypoxaemia or respiratory acidosis) Declining consciousness beyond sedation target Increasing WOB with exhaustion signs Haemodynamic instability Inability to protect airway or manage secretions Patient request
	Transition management	Plan and perform controlled intubation; continue sedative infusions during intubation and initial mechanical ventilation to ensure smooth transition and prevent awareness

## Data Availability

The data presented in this study are available on request from the corresponding author.
